# Spatial and temporal variation of malaria entomological parameters at the onset of a hydro-agricultural development in central Côte d’Ivoire

**DOI:** 10.1186/s12936-015-0871-4

**Published:** 2015-09-05

**Authors:** Nana R. Diakité, Négnorogo Guindo-Coulibaly, Akré M. Adja, Mamadou Ouattara, Jean T. Coulibaly, Jürg Utzinger, Eliézer K. N’Goran

**Affiliations:** Unité de Formation et de Recherche Biosciences, Université Félix Houphouët-Boigny, 22 BP 522, Abidjan 22, Côte d’Ivoire; Département Environnement et Santé, Centre Suisse de Recherches Scientifiques en Côte d’Ivoire, 01 BP 1303, Abidjan 01, Côte d’Ivoire; Department of Epidemiology and Public Health, Swiss Tropical and Public Health Institute, P.O. Box, 4002 Basel, Switzerland; University of Basel, P.O. Box, 4003 Basel, Switzerland

**Keywords:** *Anopheles gambiae*, *Anopheles funestus*, Malaria, Hydro-agricultural system, Transmission, Entomological parameters, Côte d’Ivoire

## Abstract

**Background:**

A deeper understanding of the ecology and small-scale heterogeneity of malaria transmission is essential for the design of effective prevention, control and elimination interventions. The spatial and temporal distribution of malaria vectors was investigated in five villages in close proximity to a hydro-agricultural system in Côte d’Ivoire over the course of construction and the early phase of irrigated rice farming.

**Methods:**

The study was carried out in five villages (Raffierkro, N’Douakro, Ahougui, Kpokahankro, Koffikro) near Bouaké, central Côte d’Ivoire, between early 2007 and late 2009. In each village, mosquitoes were collected by human landing catches and identified morphologically at genus and species level, and entomological parameters were determined. *Plasmodium* infection was assessed by dissection and an enzyme-linked immunosorbent assay.

**Results:**

A total of 19,404 mosquitoes belonging to the genus *Anopheles* were sampled during 328 human-night catches. Before the construction of the hydro-agricultural system, comparable densities of *Anopheles gambiae* were observed in all villages. In subsequent years, densities in Raffierkro and Ahougui were significantly higher than the other villages [Kruskal–Wallis (KW) test = 31.13, p < 0.001]. The density of *Anopheles funestus* in the five villages was comparable in the early stage of the project, while a high density was reported in Koffikro at the end (KW test = 11.91, p = 0.018). Transmission of *Plasmodium falciparum* is perennial in the study area. Over the course of the study, high entomological inoculation rates (EIRs) were found: 219**–**328 infectious bites per person per year with *An. gambiae*. For *An. funestus* considerably lower EIRs were observed (5.7**–**39.4). Changing patterns of *An. gambiae* were not correlated with malaria transmission.

**Conclusion:**

In this study setting, located in the bioclimatic transition zone of Côte d’Ivoire, rice cultivation was not observed to increase malaria transmission. The entomological parameters recorded until the onset of rice-growing activities in a hydro-agricultural system presented considerable heterogeneity both in space and time; a strong increase of *Anopheles* mosquitoes was observed in two of the five villages located in close proximity to the dam and irrigated rice fields. Malaria still is a main public health problem in all villages that require adequate control measures.

## Background

As a result of major droughts in sub-Saharan Africa in the 1960s and 1970s, and in order to intensify agricultural production, many Africans countries engaged in the construction of small and large dams [1, 2]. Indeed, hydro-agricultural and aquaculture developments have been realized across Africa with the aim to reduce hunger and poverty. On the one hand, this commitment lead to an improvement of agricultural production and inland fish cultivation, but on the other hand, these water resources development and management projects altered the risk of water-based and vector-borne diseases, such as schistosomiasis and malaria [3, 4].

In spite of the huge efforts across Africa to prevent, control and eliminate malaria, the disease remains a serious and complex public health issue with a heavy burden [5, 6]. Previous research has shown that ecological transformations consequential to rice developments considerably influenced the diversity and density of the culicidae fauna and sometimes malaria transmission. For example, studies carried out in Ethiopia [7, 8], Kenya [9] and Madagascar [10] suggested that irrigation enhances malaria transmission. In Mali [11] and Senegal [12] higher densities of *Anopheles* in rice area did not influence malaria transmission. The same divergent observations were made in irrigated rice farming areas in different setting in Côte d’Ivoire. Studies in the savannah area have shown that an increased density of *Anopheles* did not influence malaria transmission [13]. However, in the western forest area of Côte d’Ivoire, the high aggressive density of *Anopheles funestus* resulted in an increase of malaria transmission in villages performing one rice crop per year [14]. The three main malaria vector species in Côte d’Ivoire are *Anopheles gambiaes.s.*, *An. funestuss.s.* and *Anopheles nilis.s.* [15–18]. *Plasmodium falciparum* is the predominant malaria species encountered (80–95 % of infections), followed by *Plasmodium malariae* (7**–**10 %) and *Plasmodium ovale* (1**–**3 %) [19].

For the current study setting in central Côte d’Ivoire, located in the bioclimatic transition between the savannah in the north and tropical rainforest in the south [20], it was important to clarify the following issues. What is the effect of dam construction and irrigated rice farming on malaria transmission? Do mosquito dynamics change over the course of dam construction, including the first cycle of irrigated rice farming? Are there differences in malaria transmission parameters from one village to another in function of distance to the main dam and irrigation site? A deeper understanding of these questions at this small-scale of investigation will assist in tailoring malaria control interventions and preventive measures to the prevailing social-ecological systems.

## Methods

### Study site and characteristics

The study was carried out near Bouaké, the second largest town of Côte d’Ivoire located in the central part of the country (geographical coordinates: 7°44′N latitude, 5°41′W longitude). The mean daily temperature ranges between 23.7 and 33.8 °C. The mean annual rainfall in the years 2007–2009 ranged between 1229 and 1334 mm. The climate is tropical humid, characterized by two well-defined seasons: a dry season from November to February and a long rainy season from March to October. The vegetation is typical for the transition zone from the tropical rainforest in the south and savannah in the north.

**Fig. 1:**
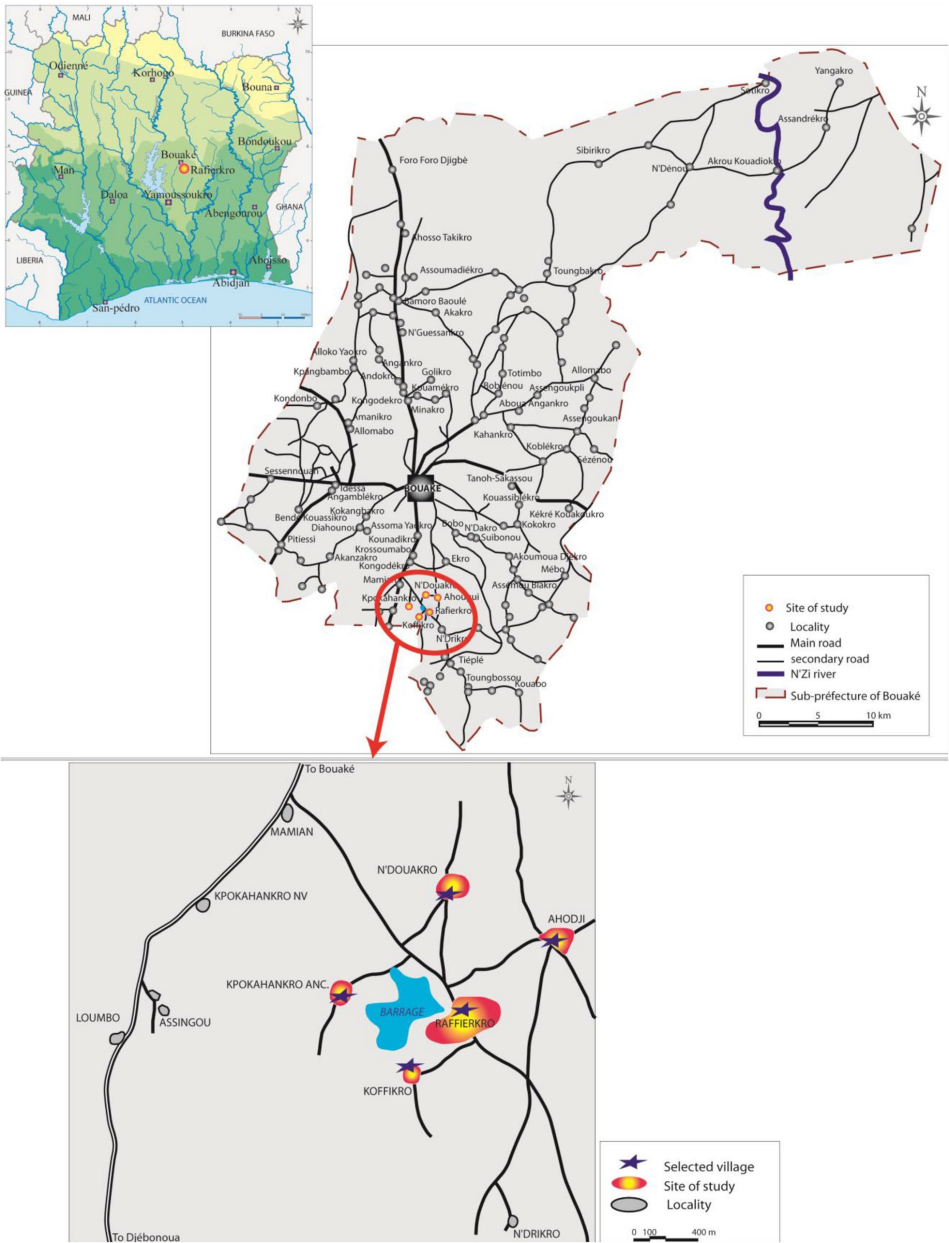
Map of the mosquito collection sites near Bouaké, central Côte d’Ivoire

The study was conducted in five villages that were all located within a maximum distance of 5 km from the Raffiekro dam site (i.e. Ahougui, Koffikro, Kpokahankro, N’Douakro and Raffierkro) located about 15 km from Bouaké. The villages are located in proximity to a small multipurpose dam in Raffierkro (Fig. 1). The five villages represent typical ecological features for central Côte d’Ivoire and show slight differences in their agricultural practices, as follows. Raffierkro formerly was a leprosy-village with a hospital. It is the most developed among the five villages with key commodities, such as electricity, water and a school with informatics class. The water availability allows for irrigation with two rice growing cycle per year. The dam was constructed on Balloba River about 100 m from the closest houses of the village. Ahougui is located at 2 km downstream of the water reservoir and at 400 m to the surface extended for the rice and fish farming. It is a traditional rice-growing village with a non-functional dam, which had previously been used for rice farming. Kpokahankro is located at 3.5 km upstream. The village is still densely forested and characterized by many streams. The predominant agricultural activities in this area are vegetable faming (e.g. tomatoes and aubergines). N’Douakro is at 1.6 km far from the dam and is a non-rice-growing village. The land is marshy and the population is mainly engaged in subsistence farming with sweetcorn, yams and cassava. Koffikro, located in close proximity to Raffierkro, is situated at 500 m away. This village has no open surface water apart from the dam. Traditionally, the main activity of the populations was the cultivation of yams and cassava. Due to the construction of the dam, people have become involved in rice and vegetable crop production.

### Study design and procedures

**Fig. 2:**
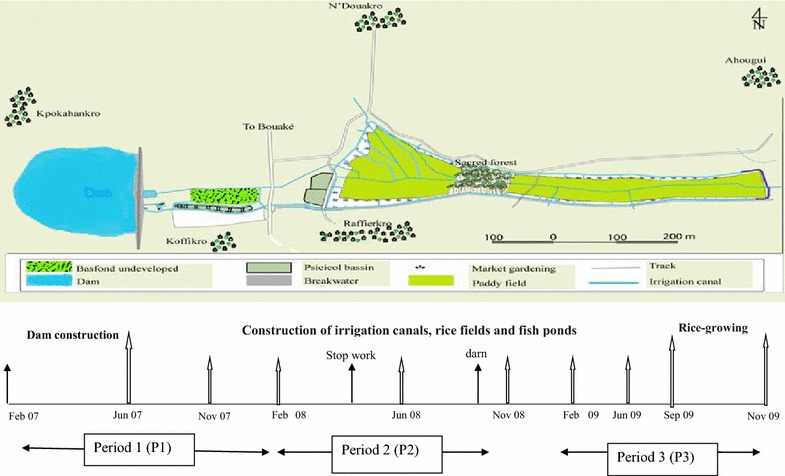
Study area showing the location of the study villages within the irrigation scheme, differents activities around the dam and month of entomological surveys

This study was designed as a repeated cross-sectional survey. There were three main study periods: (1) the construction of the dam; (2) the construction of irrigation canals, rice fields and fish ponds; and (3) the onset of rice-growing activities. The entomological surveys were done from June 2007 to November 2009, while the construction of the dam started in February 2007 (Fig. 2).

Mosquitoes were collected in four sentinel sites per village, using human landing catches [21]. Sampling was carried out in sentinel sites every month by two teams of four collectors, two indoors and two outdoors, once a month over a 9-month period. Collections were made from 18:00 to 06:00 h by the volunteers over two or three consecutive nights.

Mosquitoes caught were kept individually in haemolysis tubes plugged with cotton. All mosquitoes sampled were morphologically identified [22], sorted according to sampling site, house, date and species. *An. gambiae*, *An. funestus* and *An. nili* females were dissected to determine the degree of ovarian tracheoles coiling [23]. Salivary glands of parous mosquitoes were examined for malaria parasites using standard dissection techniques [24].

A sample of female *Anopheles* mosquitoes kept individually in 1.5 ml Eppendorf tubes and stored over silica gel were tested by enzyme-linked immunosorbent assay (ELISA) for *P. falciparum* circumsporozoite protein [25]. This test was conducted to assess the proportion of infected specimens among the main vectors species. It is assessing the transmission rate in Raffierkro’s dam site and nearby villages.

### Statistical analysis

Data were analysed using Statistica version 7.1 (Tulsa, USA). The human biting rate (HBR) was expressed as the number of female anopheline bites per person per night (b/p/n). The result was obtained by the number of anophelines captured at each sampling point divided by the total number of sampling days and the average number of collectors. Parous females percentages and survival rates using 2 days for the gonotrophic cycle were calculated [17].

The sporozoite index for a given species was calculated as the proportion of females carrying infective sporozoites in the head–thorax of the total number tested. The entomological inoculation rate (EIR) was defined as the product of human biting rate and sporozoite index. This parameter expresses the number of infectious b/p/n, and is a widely used measure of malaria transmission [26]. The Kruskal–Wallis (KW) test was used to compare the differences in mosquito densities, and HBR among villages. The χ^2^ test was used to compare sporozoite rates. For all statistics, a *p* value below 0.05 was considered as statistically significant.

### Ethical considerations

This study was approved by the institutional research commission of the Centre Suisse de Recherches Scientifiques en Côte d’Ivoire (Abidjan, Côte d’Ivoire) and, as stated before, received approval by Bouaké’s health authorities. In each village permission to work was granted by chiefs. Community members were informed in detail about the objectives, procedures, and potential risks and benefits related to the study. People who were illiterate were informed in their local language. Written informed consent was obtained at the beginning of the study. Free and informed consent was obtained from volunteer collectors and heads of household of sentinel houses. For the volunteers who served as night baits, measures had been taken to prevent malaria (i.e. prophylactic treatment). Insecticide-treated nets (ITNs) were made available for the entire population in the five villages.

## Results

### Relative importance of vector species in anopheline fauna

A total of 19,404 mosquitoes belonging to the genus *Anopheles* were sampled during 328 human-night catches. Among the entire mosquito fauna, *An. gambiae*, *An. funestus* and *An. nili* accounted for 89.7 %, while other mosquito species accounted for the remaining 10.3 %. In Ahougui, Kpokahankro and Raffierkro, the three *Anopheles* species together accounted for 92.6, 91.7 and 90.2 %, respectively. Considerably lower proportions were observed in Koffikro and N’Douakro 82.4 and 85.8 %, respectively. As shown in Table 1, the predominant *Anopheles* species in all villages was *An. gambiae* with particularly high proportions found in Raffierkro (84.6 %) and Koffikro (72.3 %).

**Table 1:** Relative abundance of *Anopheles* collected in the five villages between June 2007 and November 2009 near Bouaké central Côte d’Ivoire

Species	Village											
	Ahougui		Koffikro		Kpokahankro		N’Douakro		Raffierkro		Total	
	n	%	n	%	n	%	n	%	n	%	n	%
*An. gambiae*	4922	80.3	1919	72.3	2914	82.8	1657	76.1	4167	84.6	15,579	80.2
*An. funestus*	589	9.6	260	9.8	308	8.8	149	6.8	267	5.4	1573	8.1
*An. nili*	163	2.7	7	0.3	4	0.1	63	2.9	9	0.2	246	1.3
*An. pharoensis*	449	7.3	469	17.7	290	8.2	304	14.0	480	9.8	1992	10.3
*An. coustani*	3	0	0	0	0	0	2	0.1	0	0	5	0.03
*An. ziemanni*	1	0	0	0	1	0	1	0	0	0	3	0.02
*An. wellcomei*	3	0	0	0	2	0.1	0	0	0	0	5	0.03
*An. brohieri*	1	0	0	0	0	0	0	0	0	0	1	0.005
Total	6131	100	2655		3519	100	2176	100	4923	100	19,404	100
Total vectors	5674	92.6	2186	82.4	3226	91.7	1869	85.9	4443	90.2		

*n* number of *Anopheles* collected

In the first period of the study, 2864 *Anopheles* were collected. Among them, 2696 (94.2 %) were main malaria vector species; *An. gambiae* (68.1 %), and *An. funestus* (31.9 %). No *An. nili* were caught in the first period. In the second period, 4533 *Anopheles* were collected and 3599 (79.4 %) were malaria vectors. *An. gambiae* was the predominant species (89.0 %), followed by *An. funestus* (9.2 %) and *An. nili* (1.8 %). In the third period, 12,007 specimens were collected; among them 11,103 (92.5 %) were malaria vectors. *An. gambiae* was the predominant species (94.9 %), followed by *An. funestus* (3.4 %) and *An. nili* (1.6 %). Over the entire study period, *An.* *gambiae* was by far the most important species in Ahougui and Raffierkro, while the relative importance of *An. funestus* increased in Ahougui and Kpokahankro over the course of the current study (Table 2).

**Table 2:** Relative importance of malaria vectors collected in the five villages between June 2007 and November 2009 near Bouaké central Côte d’Ivoire

Village	Period 1		Period 2			Period 3		
	*An. gambiae*	*An. funestus*	*An. gambiae*	*An. funestus*	*An. nili*	*An. gambiae*	*An. funestus*	*An. nili*
	n (%)	n (%)	n (%)	n (%)	n (%)	n (%)	n (%)	n (%)
Ahougui	820 (44.7)	399 (46.3)	1082 (33.8)	102 (30.9)	62 (93.9)	3020 (28.7)	88 (23.0)	101 (56.1)
Koffikro	92 (5.0)	85 (9.9)	188 (5.9)	54 (16.4)	0	1639 (15.5)	121 (31.7)	7 (3.9)
Kpokahankro	240 (13.1)	153 (17.8)	679 (21.2)	87 (26.4)	3 (4.5)	1995 (18.9)	68 (17.8)	1 (0.6)
N’Douakro	186 (10.1)	102 (11,8)	191 (6.0)	25 (7.6)	1 (1.5)	1280 (12.1)	22 (5.8)	62 (34.4)
Raffierkro	497 (27.1)	122 (14.2)	1063 (33.2)	62 (18.8)	0	2607 (24.7)	83 (21.7)	9 (5.0)
Total	1835	861	3203	330	66	1,0541	382	180

*n* total number of individuals per specie

### Human biting rate

The average specific aggressive density found throughout the study area was estimated at 47.5 b/p/n with a [95 % confidential interval (CI) of 42.0**–**53.0 b/p/n] for *An.* *gambiae*, 4.8 b/p/n (95 % CI 3.9–5.6) for *An. funestus* and 0.7 b/p/n (95 % CI 0.5**–**1.0 b/p/n) for *An. nili*. The difference in the average specific aggressive density was highly significant between the three species (KW test = 540.22, p < 0.001). The *An. gambiae* aggressive density recorded over the three periods was, respectively, 19.1 b/p/n (95 % CI 14.0**–**23.7 b/p/n), 28.6 b/p/n (95 % CI 22.9**–**34.3 b/p/n) and 87.8 b/p/n (95 % CI 77.6**–**98.0 b/p/n). A significant difference was found in the aggressive density over the three periods (KW test = 136.48, p < 0.001).

The average aggressive densities evaluated for *An*. *funestus* were, 9.0 b/p/n (95 % CI 6.5**–**11.4 b/p/n), 2.9 b/p/n (95 % CI, 2.2**–**3.6 b/p/n) and 3.1 b/p/n (95 % CI 2.3**–**4.01), respectively (KW test = 7.75, p = 0.020). For *An.nili*, the average aggressive density, although low, increased significantly over the course of the study (KW test = 63.51, p < 0.001).

**Fig. 3:**
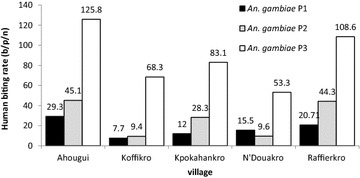
Human biting rate of *An. gambiae* (average of mosquito bites/person/night) calculated for each village according to the method (*HLC* human landing catches), in the five villages between June 2007 and November 2009

**Fig. 4:**
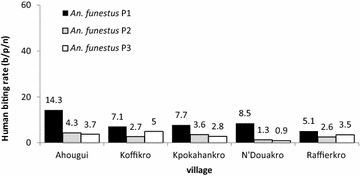
Human biting rate of *An. funestus* (average of mosquito bites/person/night) calculated for each village according to the method (*HLC* human landing catches), in the five villages between June 2007 and November 2009

**Fig. 5:**
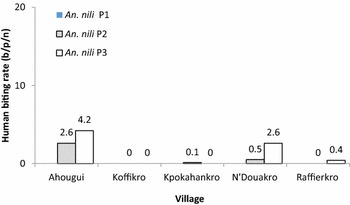
Human biting rate of *An. nili* (average of mosquito bites/person/night) calculated for each village according to the method (*HLC* human landing catches), in the five villages between June 2007 and November 2009

In the first period, similar aggressive densities were observed in all localities (KW test = 6.65, p = 0.080) with an exception in Koffikro, where a considerably lower density was observed (KW test = 12.05, p = 0.016). In Ahougui, Kpokahankro and Raffierkro, in the second period, comparable aggressive densities were found, which were significantly higher than those of N’Douakro and Koffikro (KW test = 31.13, p < 0.001). In the third period, the highest aggressive densities were observed in Ahougui (125.8 b/p/n) and Raffierkro (108.6 b/p/n), while the lowest were recorded in N’Douakro and Koffikro (Fig. 3). *An. funestus* aggressive densities within the five localities were comparable in 2007 and 2008. However, in the third period in 2009, the highest density was recorded in Koffikro (5.0 b/p/n) (KW test = 11.91, p = 0.018) (Fig. 4). For *An. nili*, the highest aggressive density was observed in Ahougui (KW test = 21.12, p < 0.001) (Fig. 5).

### Parous and daily survival rates

**Fig. 6:**
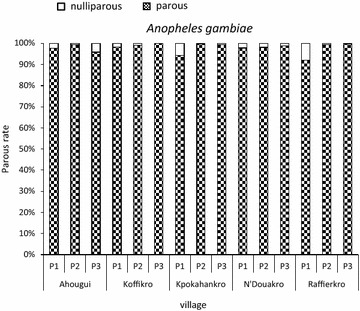
Parous and nulliparous rate of *Anopheles gambiae* in the five villages between June 2007 and November 2009

**Fig. 7:**
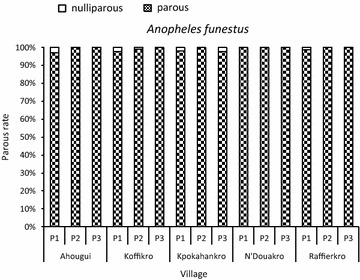
Parous and nulliparous rate of *Anopheles funestus* in the five villages between June 2007 and November 2009

During the 3-year-period, the parity rates showed a slight variation from 97.2 % (n = 1640) in Ahougui to 99.7 % (n = 668) in Koffikro. In each village, the monthly values did not vary significantly, and hence, parity rates were homogeneous. The overall parity rates for *An.s gambiae* and *An. funestus* are shown in Figs. 6 and 7. Higher parity rates of *An. gambiae* and *An. funestus* were measured in all five localities. The daily survival rate for the major malaria vector (*An. gambiae*) was very high; the values varied between 0.98 in Ahougui and 0.99 in Koffikro, Kpokahankro, N’Douakro and Raffierkro. Assuming a *P. falciparum* gonotrophic cycle of 12 days, the proportion of *An.* *gambiae* population able to transmit malaria were 82.4 % in Ahougui, 93.0 % in Koffikro and 87.6 % in Kpokahankro, N’Douakro and Raffierkro.

### Infection rate

Table 3 summarizes annual sporozoite infection rates over the course of the study. Overall, *An. gambiae* and *An. funestus* had comparable sporozoite infection rates (χ^2^ = 3.56, p = 0.059) with an exception of the first year when *An. gambiae* showed a higher infection rate than *An. funestus* (χ^2^ = 11.86, p = 0.001). The average sporozoite infection rates in *An. gambiae* changed significantly over the three-year period. These average rates were 4.7 % (95 % CI 3.4–6.2 %), 2.1 % (95 % CI 1.4–2.8 %) and 0.8 % (95 % CI 0.5–1. 2 %) in the first, second and third period, respectively. *An. funestus* infection rates during the three study periods were 1.2 % (95 % CI 0.4–2.5 %), 2.2 % (95 % CI 1–3.9 %) and 0.5 % (95 % CI 0.1–1.2 %), respectively.

**Table 3:** Annual variation of sporozoïte infection rate in the five villages near Bouaké, Central Côte d’Ivoire between June 2007 and November 2009

Village	Period 1				Period 2				Period 3			
	*An. gambiae*		*An. funestus*		*An. gambiae*		*An. funestus*		*An. gambiae*		*An. funestus*	
	N	n (%)	N	n (%)	N	n (%)	N	n (%)	N	n (%)	N	n (%)
Ahougui	348	15 (4.3)	217	2 (0.9)	448	6 (1.3)	71	1 (1.4)	891	6 (0.7)	50	0
Koffikro	62	4 (6.5)	48	0	138	3 (2.1)	137	3 (2.2)	470	1 (0.2)	284	1 (0.4)
Kpokahankro	159	5 (3.1)	114	2 (1.8)	381	15 (3.9)	75	1 (1.3)	818	7 (0.9)	43	1 (2.3)
N’Douakro	139	6 (4.3)	58	1 (1.7)	155	4 (2.6)	129	4 (3.1)	527	4 (0.8)	362	2 (0.6)
Raffierkro	298	17 (5.7)	81	1 (1.2)	477	5 (1)	51	1 (2)	916	12 (1.3)	71	0
Annual IS	1006	47 (4.7)	518	6 (1.2)	1599	33 (2.1)	463	10 (2.2)	3622	30 (0.8)	810	4 (0.5)

*N* number of *Anopheles* species examined, *n* number of *Anopheles* species infected

### Transmission

A random sample of 2411 *An. gambiae* mosquitoes were subjected to ELISA for *P. falciparum* sporozoites and identified 24 as infected. All carried *P.* *falciparum*. The average rates of *P. falciparum* infection varied from 3.6 % in Ahougui to 6.1 % in N’Douakro. These average rates obtained by ELISA were significantly higher than those obtained by dissection, but the two methods presented the same tendencies. The sub-sample tested by ELISA covered the entire period of the study. In all study villages, values obtained by ELISA testing were comparable by dissection and showed the same temporal trend.

Malaria transmission is perennial in all study villages. *An. gambiae* mosquitoes were present during the three periods with high EIRs ranging from 219 to 328 infectious bites per person per year (ib/p/y) in the three periods. During the first and third period, the peak was observed in Ahougui [459.9 (ib/p/y) and 321.2 ib/p/y]. In the second period, the highest EIR was identified at Kpokahankro (401.5 ib/p/y). In all study villages, malaria transmission estimated by *An.* *gambiae* is consistently higher than *An.* *funestus* (Z = 4.86, p < 0.001). It is provided intermittently by the two vector species (Table 4).

**Table 4:** Annual variation of entomological inoculation rate (EIR) for *An. gambiae* and *An. funestus* in five villages near Bouaké, Central Côte d’Ivoire between June 2007 and November 2009

Village	Period 1		Period 2		Period 3	
	*An. gambiae*	*An. funestus*	*An. gambiae*	*An. funestus*	*An. gambiae*	*An. funestus*
	EIR		EIR		EIR	
Ahougui	459.9	47.4	215.3	21.9	321.2	0
Koffikro	182.5	0	73	21.9	51.1	7.3
Kpokahankro	135.0	51.1	401.5	18.2	273.7	25.5
N’Douakro	244.5	51.1	91.2	10.9	156.9	3.6
Raffierkro	430.7	21.9	160.6	18.2	251.8	0
Annual EIR	327.7	39.4	219.2	23.3	256.4	5.7

## Discussion

An important aspect of malaria vector control programmes is to take into account the ecology and small-scale heterogeneity of environmental factors, in order to put forward setting-specific control measures. Studies were conducted in five villages in close proximity to a small hydro-agricultural system in an area of high malaria transmission in central Côte d’Ivoire and the dynamics of entomological parameters were studied over a three-year period.

The results shows as previously known in central Côte d’Ivoire [15, 18] that *An. gambiaes.s*, and *An. funestuss.s.* were the main malaria vectors driving the high level of transmission in this region. It was found that *An. gambiae* was the predominant species at the onset of irrigated rice farming activities in Raffierkro and Ahougui. The high density of *An. gambiae* might result from the presence of suitable larval sites, especially irrigation canals, aquaculture reservoirs and streams that were constructed during the second period. These artificial reservoirs provide suitable breeding sites for *An*. *gambiae* larvae and were linked to the exploitation of rice fields. The study site was sunlit and the irrigation water in the dug-out wells and furrows were clean, conditions which support the breeding of *An. gambiae* [27].

These observations are in agreement with previous studies done in different parts of Côte d’Ivoire, where irrigated rice farming is pursued, such as in the North, South [28, 29], and in the West of the country [30]. A study conducted in Malawi [31] also showed the proliferation of malaria vectors in rice fields.

The results of the study presented here are important for the control of mosquito larval stages in the Raffierkro area. Knowledge of larval habitat productivity could help in the planning and implementation of mosquito larval control interventions in such irrigated areas [32]. Interestingly, the density of *An. funestus* was low throughout the study. This observation could indicate that rice paddies in the current context were not favourable to larval breeding and could be the result of loss of habitats after transformation of natural habitats. Prior studies across the African continent have reported lowest densities of *An. funestus s.l.* in rice-cultivating areas [33–35].

Another aspect of the current study is that *An. gambiae* had an important implication in the transmission of malaria, in contrast to *An. funestus,* which was of lesser relevance. In Côte d’Ivoire, the three malaria vectors are *An. gambiae s.s*., *An. funestus s.s.* and *An. nili s.s.* The current results confirm prior studies conducted in the central part of Côte d’Ivoire that revealed *An. gambiae s.s.* and *An funestus s.s.* as the dominant malaria vectors [36]. *An. nili* was recorded during the two last periods of the current study, but at very low abundance, and hence it is unlikely to play a major role in malaria transmission. *Anopheles pharoensis* was not reported as vector in central Côte d’Ivoire [37], but previous studies in Senegal and Cameroon reported this vectors species there [12, 38]. Sporozoite rates recorded in the first period, before implementation of the hydro-agricultural project, were higher than in later time points. However, aggressive densities were considerably higher in all study villages during the third period, while EIR remained at the same level. High EIRs have also been reported before and might reflect the natural history of transmission during the early stage of operation of a hydro-agricultural system. This high transmission may be due to the age of female *Anopheles* mosquitoes and their capacity to transmit *Plasmodium* due to the longevity of the malaria vectors. The non-correlation of the high aggressive density with EIR in the third period indicates that in the Raffierkro area, irrigated rice farming did not result in an increase of malaria transmission. A possible explanation of this observation is that the area is in a perennial transmission zone with high longevity of malaria vectors. These results are similar to those observed in the savannah [39, 40]. However, contrasting findings were made in central Ethiopia and in the forest area of western Côte d’Ivoire, where irrigation canals, non-functional canal pools and extensive cultivation of rice resulted in favourable conditions to vector breeding sites, thus facilitating high malaria transmission [41–44]. The low transmission of malaria parasites by *An. funestus* in the five villages studied here can be justified by their low abundance. This observation underscores that *An. funestus* is a relatively rare species in irrigated rice agro-ecosystems. This study confirm previous findings that rice areas are unsuitable for *An.* *funestus* [45, 46]. Interestingly though, the current observations are in contrast with those made elsewhere in Africa [47, 48] where, after deforestation and installation of irrigated rice farming, *An.* *funestus* became the main malaria vector species with an EIR estimated at 172 ib/p/y over the study period and a high abundance (91 %); an order of magnitude higher than *An. gambiae* (7 %). During the dam construction period, insecticide-treated nets have been offered to all households in the five study villages largely subsidized. In addition, a community health worker was trained in each village and malaria treatments were made available at the village level according to a contribution equivalent to the prices observed in the public health pharmacies. The current study showed-at a scale of about 5 km around a new, multipurpose small dam that malaria transmission is characterized by small-scale heterogeneity, partially influenced by this water resource development and management system. The findings assisted in designing and prioritizing malaria control and prevention measures that are tailored to the specific social-ecological contexts. For example, villages in close proximity to the new dam require particular attention, such as high coverage with long-lasting insecticidal nets and other preventive measures particularly during the rice growing period. The investigations were performed during the construction of a dam until the first cycle of irrigated rice. It will be interesting to study patterns of transmission over a longer period to determine whether or not the results here after stabilization of rice cycles remain.

## Conclusion

During the installation of a small hydro-agricultural system until the first cycle of irrigated rice farming, heterogeneities were observed in five villages situated in close proximity to a dam. *An. gambiae* and *An. funestus* were the two prominent vectors of malaria and high transmission was primarily due to *An. gambiae.* The hydro-agricultural development is the likely cause of a large aggressive mosquito density, which did not influence the transmission of malaria in Raffierkro’s environment. However, malaria still is a main public health problem in all villages. In this setting, malaria transmission is perennial with high survival of vectors and EIRs rates. Hence adequate control measures are required. First, the future hydro-agricultural dam must be constructed far from villages. Second, malaria control can be optimized taking in account the spatial and temporal variability. Third, the vectors control in these areas with preventive measures combining long-lasting insecticidal nets (LLINs), indoor residual spraying (IRS) and larval control must be undertaken.

## Abbreviations

b/p/n: bites per person per night
CI: confidence interval
EIR: entomological inoculation rate
ELISA: enzyme-linked immunosorbent assay
HBR: human biting rate
ib/p/n: infectious bites per person per night
ib/p/y: infectious bites per person per year
ITN: insecticide-treated nets
KW: Kruskal–Wallis
LLINs: long-lasting insecticidal nets
IRS: indoor residual spraying
